# Mdwgan-gp: data augmentation for gene expression data based on multiple discriminator WGAN-GP

**DOI:** 10.1186/s12859-023-05558-9

**Published:** 2023-11-13

**Authors:** Rongyuan Li, Jingli Wu, Gaoshi Li, Jiafei Liu, Junbo Xuan, Qi Zhu

**Affiliations:** 1 College of Computer Science and Engineering, Guangxi Normal University, Guilin, China; 2 Key Lab of Education Blockchain and Intelligent Technology, Ministry of Education, Guangxi Normal University, Guilin, China; 3 Guangxi Key Lab of Multi-source Information Mining & Security, Guangxi Normal University, Guilin, China

**Keywords:** Data augmentation, Graph convolutional network, Gene expression data, WGAN-GP, Generative adversarial network

## Abstract

**Background:**

Although gene expression data play significant roles in biological and medical studies, their applications are hampered due to the difficulty and high expenses of gathering them through biological experiments. It is an urgent problem to generate high quality gene expression data with computational methods. WGAN-GP, a generative adversarial network-based method, has been successfully applied in augmenting gene expression data. However, mode collapse or over-fitting may take place for small training samples due to just one discriminator is adopted in the method.

**Results:**

In this study, an improved data augmentation approach MDWGAN-GP, a generative adversarial network model with multiple discriminators, is proposed. In addition, a novel method is devised for enriching training samples based on linear graph convolutional network. Extensive experiments were implemented on real biological data.

**Conclusions:**

The experimental results have demonstrated that compared with other state-of-the-art methods, the MDWGAN-GP method can produce higher quality generated gene expression data in most cases.

## Introduction

Over the last two to three decades, the rapid development of the genome sequencing technology has made it into reality to measure the expression level of thousands of genes from a biological sample simultaneously. Since gene expression data is extracted by various gene profiling technologies, direct reflecting the physiological state and disease of the human body [1], many computational technologies such as regression, classification and clustering can be applied on it to uncover disease mechanisms, propose novel drug targets, provide a basis for comparative genomics, and address a wide range of fundamental biological problems [2].

Nevertheless, the gene expression profile data are fundamentally limited in sample size, diversity, and the speed at which they can be gathered [3], due to the ethical challenge [4] and high expenses of money for gathering gene expression data through biological experiments. For example, the per person costs were US$604-1932 for exome sequencing, and US$2006-3347 for whole genome sequencing in 2018 [5]. In addition, much bias or noise, which results from the errors in the splicing process of short reads [6] and various batch effects [7], makes it a great challenge to take advantage of the gene expression data effectively. Therefore, it is desired to generate biologically plausible synthetic gene expression data, which can be applied in such downstream tasks as marker gene detection, cell type clustering, gene association identification, cancer stages prediction, and so on [3]. In recent years, data augmentation (DA) methods, being capable of enriching data sets, mitigating data imbalance and data noise issues, have been extensively studied in the area of generating synthetic gene expression data.

To the best of our knowledge, there are generally three categories of data augmentation methods for generating gene expression data, such as sample-based, simulator-based, and generative adversarial network-based. The sample-based methods include random sampling [8], mean sampling [9], resampling [10], and oversampling [11, 12], which are prone to the problem of overfitting [13] or distribution marginalization [14]. The simulator-based methods [15, 16] generate synthetic transcriptomics datasets based on known regulatory networks. Since they perform similarly to the random simulators [2], the key features of gene expression data can not be simulated [17]. With the rapid development of deep learning technology, the Generative Adversarial Network (GAN)-based method, being able to produce more diverse and higher quality samples than the former two methods, has received major attention [1, 2]. It is also studied in this paper.

In 2020, Chaudhari et al. [18] firstly proposed modified generator GAN (MG-GAN), which is fed with original data along with minimalistic multivariate noise to generate data with Gaussian distribution. In 2021, Kwon et al. [19] indicated that GANs are not effective with whole genes, and expanded RNA expression data for selected significant genes using GANs. Both of the two methods adopt the original unconditioned generative model, which has no control on modes of the data being generated [20]. In 2022, Ahmed et al. [21] developed method omicsGAN to integrate two omics data and their interaction network into a Wasserstein Generative Adversarial Network (WGAN) [22]. Nevertheless, gradient explosion is common when training WGAN. In 2020, Marouf et al. [23] adopted conditional single-cell generative adversarial neural networks (cscGAN) to produce single-cell RNA-seq data. It learns non-linear gene-gene dependencies from complex, multiple cell type samples and uses this information to generate realistic cells of defined types. In 2022, Han et al. [1] put forward the method Gene-CWGAN, which stabilizes the distribution of generated samples with a dataset partition method, and adopts constraint penalty term to improve the diversity of generated samples. In the same year, Viñas et al. [2] proposed a new simulator (it is called as S-WGAN-GP in this paper) based on WGAN-GP (Wasserstein Generative Adversarial Network with Gradient Penalty) [24]. S-WGAN-GP concatenates the sample covariates with the input features and samples the class labels from the real distribution. The S-WGAN-GP simulator can be used at a higher scale to produce tissue- and organ-specific transcriptomics data.

In the process of training generative adversarial networks, mode collapse is a serious issue to be concerned about. It may be an effective channel to alleviate the problem to improve the diversity of training samples as well as feedback signals. Among the above mentioned approaches, the diversity of feedback signals may be constrained for just one discriminator being adopted in the GANs. Therefore, in this paper, the collaboration of multiple discriminators is explored. The main contributions of this paper are summarized as follows:

1. The multiple discriminator WGAN-GP (MDWGAN-GP) model is proposed. It can ensure the high quality of the generated gene expression data. Multiple discriminators are adopted prevent mode collapse via providing more feedback signals to the generator.
2. A novel approach based on linear graph convolutional network (GCN) is put forward to enrich training samples, avoiding over-fitting or mode collapse caused by small sample size in high dimensional data.
3. The pan-cancer gene expression datasets were produced to demonstrate the effectiveness of the MDWGAN-GP approach. A data preprocess method is conducted to select the genes with high confidence or top ranking from protein-protein interaction networks, so as to relieve the curse of dimensionality encountered in the training. Extensive experiments were implemented to compare the quality of generated gene expression data between the MDWGAN-GP method and other state-of-the-art ones.

## Preliminaries

### Conditional generative adversarial network

The conditional generative adversarial network (CGAN) [20] attempts to generate samples of specified labels through input labels and noise. As the normal generative adversarial network (GAN) [25], a CGAN model consists of a generation network *G* and a discrimination network *D*. Given some noise *z* and conditional information *y* (e.g. category labels, data with different modalities), the generator *G* learns to produce synthetic samples similar to the real distribution. The discriminator D needs to distinguish whether the input sample is from authentic sample *p*(*x*) or from sample *p*(*z*) produced by the generator *G*. The loss function of CGAN can be formulated as:

1 $$\begin{aligned} \mathop {\min }\limits _G \mathop {\max }\limits _D V(D,G) = {E_{x\sim {p{(x)}}}}[\log D(x|y)] + {E_{z\sim {p{(z)}}}}[\log (1 - D(G(z|y)|y))] \end{aligned}$$

### Conditional Wasserstein generative adversarial network with gradient penalty

Different from CGAN, the Wasserstein generative adversarial network (WGAN) [22] tries to generate samples with just input noise. It applies the Wasserstein distance instead of the Jensen-Shannon (JS) divergence to evaluate the distribution distance between the real samples and the generated ones, making the training process more stable and faster than the normal generative adversarial network. The Wasserstein generative adversarial network with gradient penalty (WGAN-GP) [24] is an modified model based on WGAN, penalizing the norm of gradient of the discriminator with respect to its input. In 2020, Zheng et al. [26] further improved the WGAN-GP model from the addition of conditional information and proposed the CWGAN-GP model, whose loss function can be formulated as:

2 $$\begin{aligned} \begin{aligned} \mathop {\min }\limits _G \mathop {\max }\limits _D V(D,G) = {E_{x\sim {p{(x)}}}}[D(x|y)] - {E_{z\sim {p{(z)}}}}[D(G(z|y)|y)]+\\ \lambda {E_{{\hat{x}}\sim {p{({{\hat{x}}})}}}}[{(||{\nabla _{{\hat{x}}}}D({{\hat{x}}}|y)|{|_2} - 1)^2}], \end{aligned} \end{aligned}$$

where ${E_{{\hat{x}}\sim {p{({{\hat{x}}})}}}}[{(||{\nabla _{{\hat{x}}}}D({{\hat{x}}}|y)|{|_2} - 1)^2}]$ is the gradient penalty term.

### Graph convolutional network

The emerging graph convolutional networks (GCNs) [27–29] are able to extract well spatial correlation in non-Euclidean structures and maintain shift-invariance. Let *G*=(*V*, *E*) be an undirected graph, where *V* and *E* represent the set of nodes $v_{i}$ $\in$ $V$ (*i*=1,2,...,*n*) and edges ($v_{i}$,$v_{j}$)$\in$ $E$, respectively. $A$ $\in$ $R^{n\times n}$ is the adjacent matrix of *G*, where $A_{ij}$ indicates whether there is an edge between $v_{i}$ and $v_{j}$, or the similarity between them basing on a similarity measure. Let $H^{(l)}$ represent the graph node representations at the *l*-th ($l$ $\in$ $N$) layer, the propagation rule for calculating the graph node representations at the $(l+1)$-th layer is formulated as:

3 $$\begin{aligned} H^{(l+1)}=f\left( {\widetilde{D}}^{-\frac{1}{2}} \widetilde{A}{\widetilde{D}}^{-\frac{1}{2}}H^{(l)}W^{(l)}\right) , \end{aligned}$$

where *f*($\cdot$) is a no-linear activation function, $\widetilde{A}$=*A*+*I*, and $W^{(l)}$ is the weight matrix of the *l*-th layer. ${\widetilde{D}}^{-\frac{1}{2}}\widetilde{A}{\widetilde{D}}^{-\frac{1}{2}}$ is a symmetric normalized Laplacian matrix, where ${\widetilde{D}}_{ii}$=$\sum _{j=1}^{n}{\widetilde{A}}_{ij}$.

## Proposed method

Recently, Viñas et al. [2] proposed a WGAN-GP based simulator S-WGAN-GP to generate specific tumour gene expression data. Though conditional restrictions are added, model collapse or over-fitting may not be exempted for small training samples due to just one discriminator is adopted. In addition, some inherent defects are also harboured in WGAN-GP, such as training unstable and failing to generate diverse samples [1, 30]. Therefore, in this section, an improved data augmentation approach, the multiple discriminator WGAN-GP (MDWGAN-GP) model, is proposed. We begin with enriching the training samples with linear graph convolution [31, 32], then a generative adversarial network with multiple discriminators is devised based on WGAN-GP. The concrete descriptions are as follows. The source code of method MDWGAN-GP can be downloaded from https://github.com/lryup/MDWGAN-GP.

### Enriching training samples

It is generally regarded that enriched training samples contribute to GAN capturing the original distribution [33]. Inspired by methods exerted on image data to enrich training samples, i.e., rotation, flipping, and cropping, a novel approach suitable for gene expression data is proposed. Given a raw gene expression matrix $X_1$ with *n* rows (samples) and *m* columns (genes), where each entry represents the expression level of a given gene in a particular sample. A pair of *K*-Nearest Neighbors (KNN) graphs [34, 35] $G_E$ and $G_C$ are built from matrix $X_1$ based on Euclidean distance and Cosine distance, respectively. Each vertex of them denotes a sample, and the edge demonstrates that there is a strong relationship between the connected two samples. Linear graph convolution is performed to update the vertices (samples), i.e., aggregating the information of their neighbor ones. The updated gene expression matrices $X_2$ and $X_3$ are depicted as follows:

4 $$\begin{aligned} X_2= & {} f\left( {\widetilde{D}_E}^{-\frac{1}{2}}\widetilde{A}_ E{\widetilde{D}_E}^{-\frac{1}{2}}X_1\right) , \end{aligned}$$

5 $$\begin{aligned} X_3= & {} f\left( {\widetilde{D}_C}^{-\frac{1}{2}}\widetilde{A}_ C{\widetilde{D}_C}^{-\frac{1}{2}}X_1\right) , \end{aligned}$$

where *f*($\cdot$) is a linear activation function. $\widetilde{A}_E$=$A_E$+*I* (resp. $\widetilde{A}_C$=$A_C$+*I*), where $A_E$ and $A_C$ are the adjacency matrices of graphs $G_E$ and $G_C$, respectively. ${\widetilde{D}}_E{_{ii}}$=$\sum _{j=1}^{n}{\widetilde{A}}_E{_{ij}}$, ${\widetilde{D}}_C{_{ii}}$=$\sum _{j=1}^{n}{\widetilde{A}}_C{_{ij}}$.

### Adversarial simulator for augmenting gene expression data

**Fig. 1:**
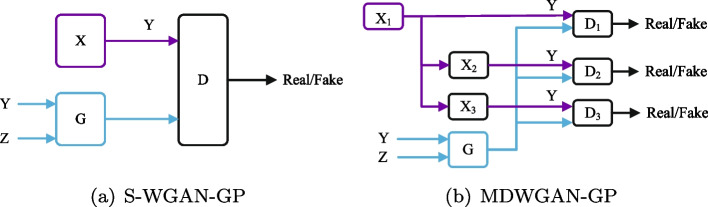
the structures of the S-WGAN-GP model and the MDWGAN-GP model

It has been regarded that the adoption of multi discriminators can improve the stability of optimization process [33]. In this subsection, an adversarial simulator MDWGAN-GP with three discriminators is devised, as shown in Fig. 1.

Figure 1a shows the S-WGAN-GP model, and Fig. 1b illustrates the structure of MDWGAN-GP proposed in this paper. In the MDWGAN-GP model, the distribution of the original data are expected to be learned from two updated gene expression matrices $X_2$ and $X_3$ besides raw gene expression matrix $X_1$. Hence two more discriminators $D_2$ as well as $D_3$ are added and fed with $X_2$ and $X_3$, respectively. Nevertheless, it is worth noticed that the generator is still anticipated to learn from the raw samples $X_1$ principally rather than the updated ones, which play auxiliary roles in the process of training.

#### The objective function

In a generative adversarial network, the generator tries to produce samples that look real enough to trick the discriminator, while the discriminator attempts to distinguish the generated samples from the real ones. Here the objective functions are designed for one generator and three discriminators in MDWGAN-GP, as illustrated in Equation (6):

6 $$\begin{aligned} \begin{aligned} V(D_i,G) = {E_{X_i\sim {p{(X_i)}}}}[D_i(X_i|Y)]-{E_{Z\sim {p{(Z)}}}}[D_i(G(Z|Y)|Y)]+\\ \lambda {E_{{\hat{X}}_i\sim {p{({{\hat{X}}_i})}}}}[{(||{\nabla _{{\hat{X}}_i}}D_i({{\hat{X}}_i |Y})|{|_2} - 1)^2}], i=1,2,3, \end{aligned} \end{aligned}$$

where *Y* indicates the conditional labels. $\lambda$ is a hyperparameter determining strength of gradient penalty ${E_{{\hat{X}}_i\sim {p{({{\hat{X}}_i} )}}}}[{(||{\nabla _{{\hat{X}}_i}}D_i({{\hat{X}}_i|Y})|{|_2} - 1)^2}]$. $X_i$ is the real samples, *Z* denotes the noise samples, $\hat{X_i}$ represents the samples randomly chosen from the real ones or the generated ones. The whole optimization objective functions of generator and discriminator are formulated as Equation (7) and Equation (8):

7 $$\begin{aligned}{} & {} {\mathop {\mathrm{{min}}}\limits _G V( {D_1,D_2,D_3,G}) = V({D_1,G}) + \frac{{{\lambda _g}}}{{2}}[V(D_2,G)+V(D_3,G)]}, \end{aligned}$$

8 $$\begin{aligned}{} & {} \mathop {\mathrm{{max}}}\limits _{D_1,D_2,D_3} V({D_1,D_2,D_3,G})=V({D_1,G})+\frac{{{\lambda _d}}}{{2}}[V(D_2,G)+V(D_3,G)], \end{aligned}$$

where ${\lambda _g}$ and ${\lambda _d}$ denote two small adjustable parameters assisting model learning. All discriminators are trained through weight sharing to improve model performance [33].

### Architecture

**Fig. 2:**
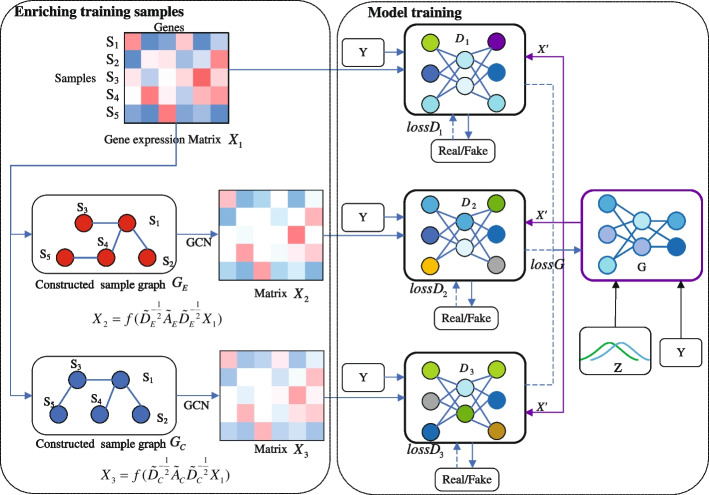
The structure diagram of MDWGAN-GP model

Figure 2 shows the architecture of the proposed simulator MDWGAN-GP. The generator *G* receives noise vector *Z* and conditional label *Y* as input and produces vector $X'$ of synthetic expression values. The discriminator $D_i$ (*i*=1,2,3) takes either a real gene expression sample $X_i$ or a synthetic sample $X'$, in addition to a conditional label *Y*, and tries to distinguish whether the input sample is real or fake. Matrices $X_2$ and $X_3$ are respectively produced with a linear graph convolution of sample graphes $G_E$ and $G_C$, which are respectively constructed from matrix $X_1$ based on Euclidean distance and Cosine distance.

## Experimental details

The effectiveness of MDWGAN-GP is verified through extensive experiments. We began with comparing the model performances of CGAN [20], CWGAN [36], CWGAN-GP [26], Gene-CWGAN [1], S-WGAN-GP [2], and MDWGAN-GP with the similarity *dist*($\cdot$, $\cdot$) on fifteen datasets, and the diversity of samples generated by these models through sample dimension visualization. Then we compared the model performances with the classification ability of generated samples. Next, we compared the performances among these models in terms of the correlations among key genes. Finally, we compared the differentially expressed genes, identified using the generated datasets, with those identified using the real ones.

### Data preparation and parameter settings

In the experiments, real biological datasets are acquired from four databases:

(1) The Cancer Genome Atlas (TCGA). It is a public biospecimen repository which aims to augment the understanding of the molecular mechanisms of cancers. The database contains high-throughput genomic data from over 20,000 primary cancer and matched healthy samples spanning 33 cancer-types.

(2) The Genotype-Tissue Expression (GTEx). It is also a public resource built to study tissue-specific gene expression and regulation. It contains samples collected from 54 non-diseased tissue sites across nearly 1000 individuals [37].

(3) The String dataset. String is a database which records known and predicted protein-protein interactions, including physical as well as functional connections. The latest Human Protein Interaction Network version 11.5 was adopted in the experiments.

(4) The HumanNet dataset. HumanNet [38] is a database that covers 99.8% of human protein-coding genes. The latest functional gene network (HumanNet-FN) version 3 [39] was adopted in the experiments.

The data preparation was conducted was follows. Firstly, the raw RNA-seq sample datasets of TCGA and GTEx were acquired from Wang et al. [40]. Fifteen common tissues between TCGA and GTEx datasets were selected to construct the GT dataset, which consisted of 9,147 samples and 18,154 genes. Secondly, the String PPI network were consisted of 11,938,499 edges and 19,385 proteins, and 360,783 edges as well as 14,220 proteins were retained through filtering out the edges with a score less than 800. The transfer from protein ID to gene ID, then to gene name was conducted with the Genome Reference Consortium Human Build 38 Organism (GRCH38) database, and R packages AnnotationDbi and org.Hs.eg.db. Then 13,035 genes were remained by dropping duplicate ones, for some proteins correspond to multiple genes. Thirdly, among the 977,495 edges and 18,458 genes of HumanNet, 15,443 genes and 97,749 edges were left by choosing the top 10% more reliable edges. Finally, the genes that were not belong to the String or the HumanNet PPI networks were dropped from the GT dataset, and 9147 samples and 10612 genes were remained. Both logarithmic transformation and z-score were adopted to normalize the gene expression values. The number of samples of the fifteen common tissues were illustrated in Table 1.

**Table 1:** The number of samples of the fifteen common tissues

Tissue	GTEx	TCGA	Normal	Cancer	Total Samples
Bladder	11	379	28	362	390
Breast	89	1092	199	982	1181
Cervix	11	261	13	259	272
Colon	339	423	390	372	762
Esophagus_gas	150	0	150	0	150
Esophagus_muc	267	0	267	0	267
Esophagus_mus	242	194	253	183	436
Kidney	32	897	158	771	929
Liver	115	383	172	326	498
Lung	313	1102	423	992	1415
Prostate	106	474	154	426	580
Salivary	55	502	97	460	557
Stomach	192	413	225	380	605
Thyroid	318	494	371	441	812
Uterus	82	211	105	188	293
Counts	2322	6825	3005	6142	9147

In the experiments, 10% of the samples in all datasets were randomly selected as the training set, while the 90% rest ones were as the test set. Both the generator and the discriminator models included two layers of fully connected hidden layers, each of which had 256 nerves. The hidden layer adopted the ReLU activation function, and the output layer did not use any. The RMSProp optimizer was executed with a learning rate of 0.0005 [41]. Some hyperparameters were set as follows: $\lambda$=10 [24], $\lambda _g$=0.2, and $\lambda _d$=0.02 [33]. The training process was terminated when the validation score *dist*($D^X$, $D^Z$) was not improved for 20 consecutive times, or it reached the maximum iterations of 500.

### Evaluation index

In this section, evaluation indexes for estimating the performance of generative model are described. Assume that $X_{m_1\times n}$ and $Z_{m_2\times n}$ are a pair of matrices recording real and synthetic gene expression observations, respectively. The rows of them respectively denote a set of $m_1$ real cancer samples and $m_2$ synthetic ones, the columns of them denote a set of *n* genes, and the entries of them are real numbers, i.e., $x_{ij}$, $z_{ij}$ $\in$ $R$. Let $D^X$ and $D^Z$ be a pair of $n$ $\times$ $n$ symmetric matrices corresponding to *X* and *Z*. In matrix $D^X$ (resp. $D^Z$), each entry $d_{jk}^{X}$ (resp. $d_{jk}^{Z}$) records the pairwise distance between the *j*-th and the *k*-th genes, i.e., the pearson correlation coefficient between columns $x_{-j}$ (resp. $z_{-j}$) and $x_{-k}$ (resp. $z_{-k}$), as defined in Equation (9) (resp. Equation (10)):

9 $$\begin{aligned} \begin{array}{*{20}{c}} {d_{jk}^{X}=\frac{\mathop \sum \limits _{i=1}^{m_1}(x_{ij} -{\bar{x}}_{-j})\mathop \sum \limits _{i=1}^{m_1} (x_{ik}-{\bar{x}}_{-k})}{ \sqrt{\mathop \sum \limits _{i=1}^{m_1}(x_{ij} -{\bar{x}}_{-j})^2}\sqrt{\mathop \sum \limits _{i=1}^{m_1} (x_{ik}-{\bar{x}}_{-k})^2}} } \end{array} \end{aligned}$$

10 $$\begin{aligned} \begin{array}{*{20}{c}} {d_{jk}^{Z}=\frac{\mathop \sum \limits _{i=1}^{m_2}(z_{ij} -{\bar{z}}_{-j})\mathop \sum \limits _{i=1}^{m_2} (z_{ik}-{\bar{z}}_{-k})}{ \sqrt{\mathop \sum \limits _{i=1}^{m_2}(z_{ij} -{\bar{z}}_{-j})^2}\sqrt{\mathop \sum \limits _{i=1}^{m_2} (z_{ik}-{\bar{z}}_{-k})^2}} } \end{array} \end{aligned}$$

where $${\bar{x}}_{-j}$$=$$\frac{\sum \limits _{i=1}^{m_1}x_{ij}}{m_1}$$, $${\bar{x}}_{-k}$$=$$\frac{\sum \limits _{i=1}^{m_1}x_{ik}}{m_1}$$, $${\bar{z}}_{-j}$$=$$\frac{\sum \limits _{i=1}^{m_2}z_{ij}}{m_2}$$, $${\bar{z}}_{-k}$$=$$\frac{\sum \limits _{i=1}^{m_2}z_{ik}}{m_2}$$.

Let *dist*($D^X$, $D^Z$) represent the similarity between matrices $D^X$ and $D^Z$, measuring whether the pairwise correlation between genes from the real data are correlated with those from the synthetic data, as defined in Equation (11) [2]:

11 $$\begin{aligned} \begin{array}{*{20}{c}} dist(D^X, D^Z)=\sum \limits _{i=1}^{n-1}\sum \limits _{j=i+1}^n(\frac{d^X_{ij} -\mu (D^X)}{\sigma (D^X)})(\frac{d^Z_{ij}-\mu (D^Z)}{\sigma (D^Z)}), \end{array} \end{aligned}$$

where $\mu (D^X)$ and $\sigma (D^X)$ are defined as Equation (12) and Equation (13), and $\mu (D^Z)$ and $\sigma (D^Z)$ are defined accordingly.

12 $$\begin{aligned} \begin{array}{*{20}{c}} {\mu (D^X)=\frac{2}{n(n-1)}\sum \limits _{i=1}^{n-1}\sum \limits _{j=i+1}^n d^X_{ij}} \end{array} \end{aligned}$$

13 $$\begin{aligned} \begin{array}{*{20}{c}} {\sigma (D^X)=\sqrt{\frac{2}{n(n-1)}\sum \limits _{i=1}^{n-1}\sum \limits _{j=i+1}^n {(d^X_{ij}-\mu (D^X))}^2}} \end{array} \end{aligned}$$

In addition, the classification performance obtained by taking advantage of the synthetic gene expression data is also adopted to measure the performance of generative model, as depicted from Equation (14) to Equation (18):

14 $$\begin{aligned} Accuracy= & {} \frac{TP+TN}{TP+FP+FN+TN} \end{aligned}$$

15 $$\begin{aligned} Precision= & {} \frac{TP}{TP+FP} \end{aligned}$$

16 $$\begin{aligned} Recall= & {} \frac{TP}{TP+FN} \end{aligned}$$

17 $$\begin{aligned} F1-score= & {} \frac{2\times Precision\times Recall}{Precision+Recall} \end{aligned}$$

18 $$\begin{aligned} Mcc= & {} \frac{TP\times TN-FP\times FN}{\sqrt{(TP+FN)\times (TP+FP)\times (TN+FN) \times (TN+FP)}} \end{aligned}$$

where TP (resp. TN) denotes the number of positive (resp. negative) samples correctly labeled by the classifier. FP (resp. FN) represents the number of negative (resp. positive) samples incorrectly labeled as positive (resp. negative) ones. Mcc denotes Matthews correlation coefficient.

### Comparison of similarity *dist*($\cdot$, $\cdot$) of different models

In Table 2, the performance of similarity *dist*($\cdot$, $\cdot$) is compared among different models. For each dataset, the generated sample set has the same size as the corresponding test set. From this table we can see that the presented model MDWGAN-GP outperforms other models in 11 of the 15 datasets. Its average *dist*($\cdot$, $\cdot$) among all of the datasets is 0.704, which is apparently higher than those of other five models.

**Table 2:** Comparisons of similarity between the real and generated samples

Tissues	CGAN	CWGAN	CWGAN-GP	Gene-CWGAN	S-WGAN-GP	MDWGAN-GP
Bladder	0.008	0.432	**0.641**	0.603	0.594	0.596
Breast	0.011	0.437	0.766	0.763	0.759	**0.788**
Cervix	0.013	0.438	0.521	0.534	0.503	**0.550**
Colon	0.007	0.392	0.791	0.844	0.831	**0.853**
Esophagus_gas	0.004	0.204	0.387	0.500	0.462	**0.511**
Esophagus_muc	0.007	0.300	0.407	0.469	0.479	**0.489**
Esophagus_mus	0.008	0.386	0.810	**0.820**	0.748	0.818
Kidney	0.008	0.378	0.773	0.777	0.773	**0.813**
Liver	0.006	0.334	0.643	0.740	**0.758**	0.731
Lung	0.008	0.416	0.750	0.749	0.751	**0.754**
Prostate	0.011	0.362	0.687	**0.742**	0.728	0.739
Salivary	0.011	0.393	0.570	0.604	0.599	**0.648**
Stomach	0.007	0.453	0.759	0.766	0.750	**0.774**
Thyroid	0.008	0.296	0.730	0.771	0.792	**0.795**
Uterus	0.008	0.422	0.673	0.629	0.666	**0.706**
Average	0.008	0.376	0.660	0.687	0.680	0.704

Bold value indicates the best result acquired for a certain tissue

**Fig. 3:**
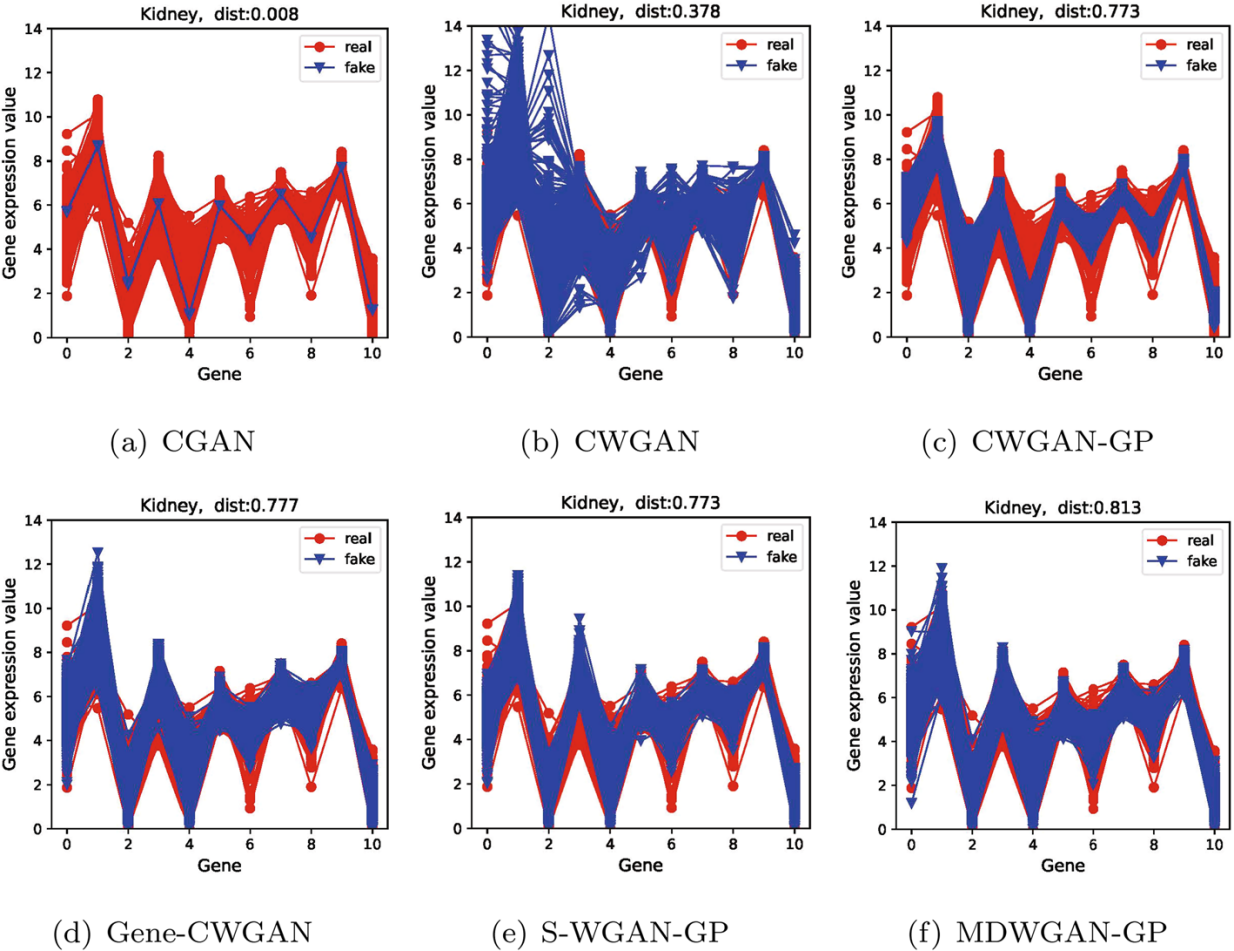
The real and the generated distribution plots of the kidney dataset

**Fig. 4:**
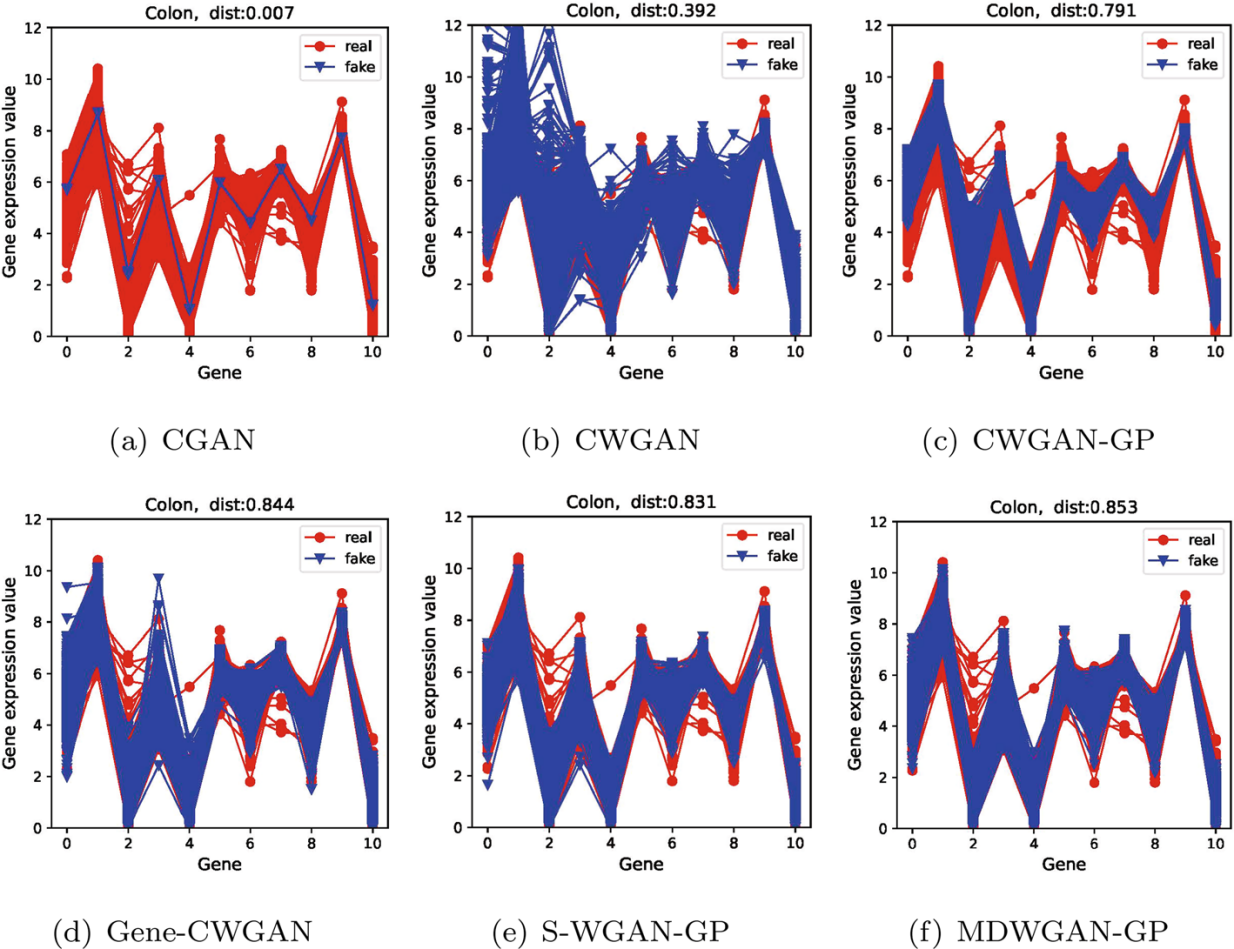
The real and the generated distribution plots of the colon dataset

**Fig. 5:**
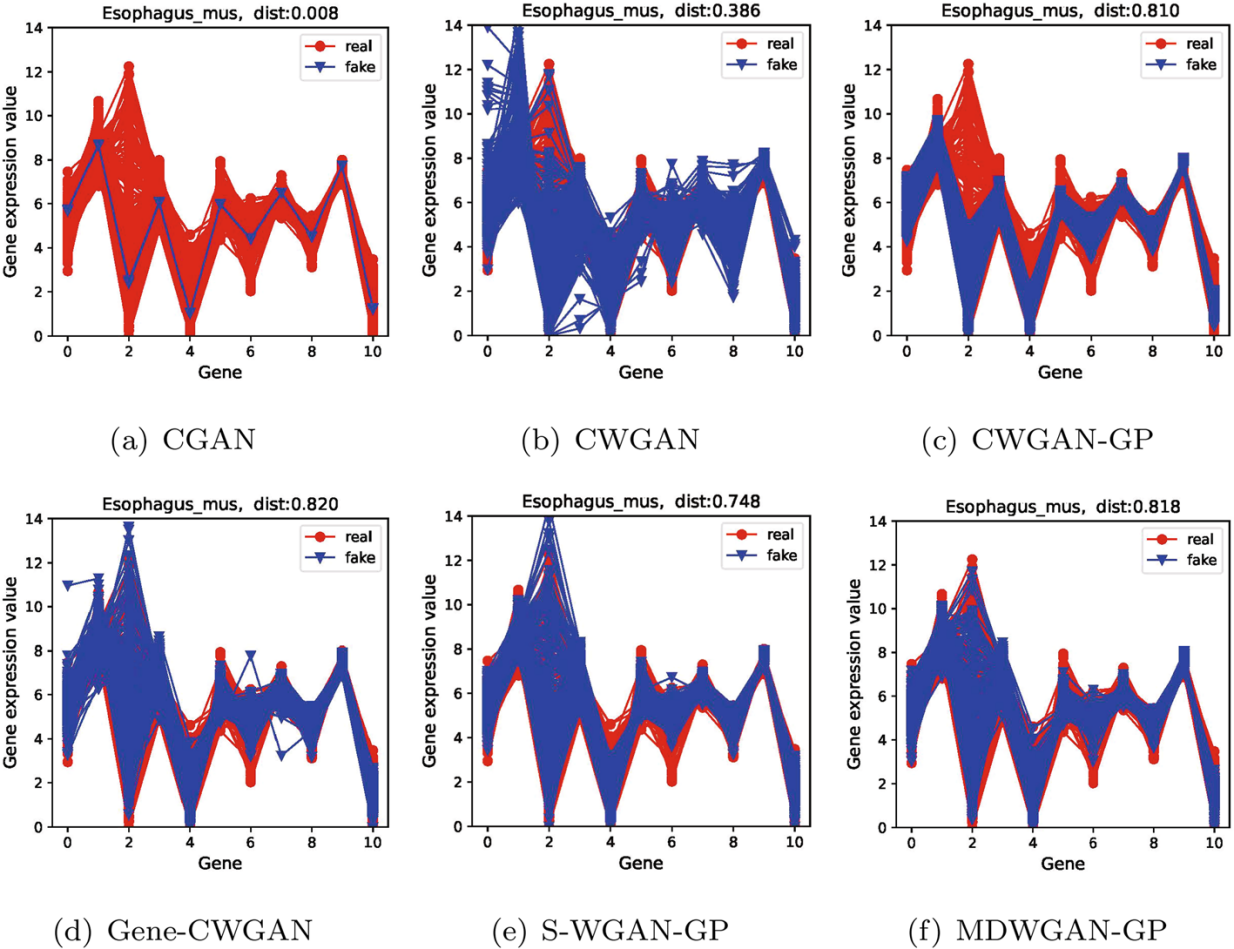
The real and the generated distribution plots of the esophagus_mus dataset

In addition, as shown in Figs. 3 , 4 and  5, the comparisons of distributions are demonstrated between the generated samples and the real samples for the first 11 genes, reflecting intuitively the diversity of generated samples. In all figures, the horizontal coordinates indicate the number of genes, and the vertical ones denote the gene expression values. The red line represents the real samples, and the blue one represents the generated samples.

From Figs. 3 , 4 and  5 we can see that compared with the samples generated by the other five models, those generated by model MDWGAN-GP generally have distributions more similar to the real samples. The samples generated by model CGAN concentrate in a very narrow range, indicating that original data distribution and the generated data distribution hold a negligible overlapping area, for JS divergence adopted by model CGAN may lead to gradient disappearance and mode collapse [42]. CWGAN adopts Wasserstein distance to solve the problem of mode collapse. However, it generates samples deviating from the original values due to gradient explosion resulting from the absence of gradient penalty [24]. CWGAN-GP avoids gradient explosion effectively with the addition of gradient punishment. Nevertheless, because the true value range of each feature is unknown and the output layer activation function of CWGAN-GP forcibly limits the generation space [1], the diversity of its samples remains poor at the distribution margins. Gene-CWGAN expands the generation space of the generation model by removing the tanh activation function of the CWGAN-GP generation model, and avoids the expansion of learning fluctuation with a constraint penalty term [1]. Nevertheless, the generated samples may deviate from the original ones. As shown in Fig. 4d, the maximum original values of the 0-th and the 3-th genes are respectively 7 and 8, while the maximum generated values of them are respectively close to 9 and 10. Similar to Gene-CWGAN, S-WGAN-GP also expands the generation space by removing the tanh activation function, and it can generate sample data with specified conditions. In order to further improve model stability and the diversity of generated samples, enriched training samples are produced with the aid of multiple discriminators in the MDWGAN-GP method. As shown in Fig. 3 , 4 and  5, the samples generated by MDWGAN-GP have more satisfying diversity at the distribution margins.

### Comparison of classification ability of samples generated based on different models

As illustrated in Tables 3, 4 and 5, the classification ability of generated samples is evaluated in terms of classifying the normal and the cancer samples. In the experiments, three kinds of classical classification methods, i.e., random forests (RF) [43], *K*-nearest neighbors (KNN) [44], and multi layered perceptron (MLP) [45], were adopted. The number of trees $n_{estimators}$ was 200 for RF, the number of neighbours *K* was 5 for KNN, and two hidden layers with 128 units and the ReLU activation function were adopted for MLP. For each method, the average results of ten runs are calculated and presented. It can be seen from the three tables that among the three methods the samples generated with the MDWGAN-GP model perform the best classification ability in the vast majority of cases. Furthermore, basing on the classification methods RF and KNN, the samples generated with the MDWGAN-GP model even present superior classification performance than the real samples (denoted as “Real” in the three tables).

**Table 3:** Comparisons of classifying the normal and the cancer samples (Accuracy%)

Methods	Real	CGAN	CWGAN	CWGAN-GP	Gene-CWGAN	S-WGAN-GP	MDWGAN-GP
RF	97.63	39.47	55.49	96.70	97.29	97.72	**97.74**
KNN	96.91	64.04	64.61	96.68	97.11	**97.21**	97.20
MLP	98.55	48.87	59.56	97.54	97.64	97.75	**97.82**

Bold value indicates the best result acquired for a certain tissue

**Table 4:** Comparisons of classifying the normal and the cancer samples (F1-score%)

Methods	Real	CGAN	CWGAN	CWGAN-GP	Gene-CWGAN	S-WGAN-GP	MDWGAN-GP
RF	97.62	32.30	54.97	96.72	97.28	97.72	**97.74**
KNN	96.91	55.23	57.10	96.69	97.12	**97.21**	**97.21**
MLP	98.56	49.42	58.51	97.55	97.64	97.76	**97.83**

Bold value indicates the best result acquired for a certain tissue

**Table 5:** Comparisons of classifying the normal and the cancer samples (Mcc%)

Methods	Real	CGAN	CWGAN	CWGAN-GP	Gene-CWGAN	S-WGAN-GP	MDWGAN-GP
RF	94.61	2.17	-2.39	92.76	93.83	94.83	**94.89**
KNN	92.99	-1.37	1.97	92.59	93.49	93.67	**93.68**
MLP	96.73	-11.88	5.02	94.52	94.70	94.97	**95.12**

Bold value indicates the best result acquired for a certain tissue

**Fig. 6:**
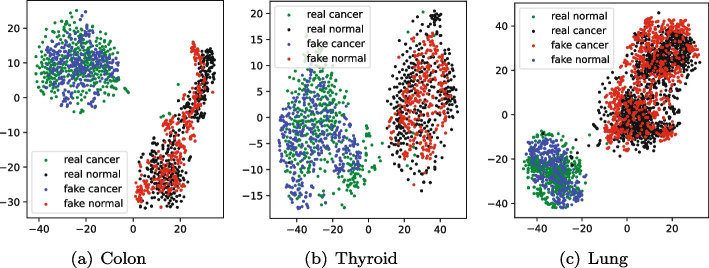
The overlap of the real and the generated samples

**Fig. 7:**
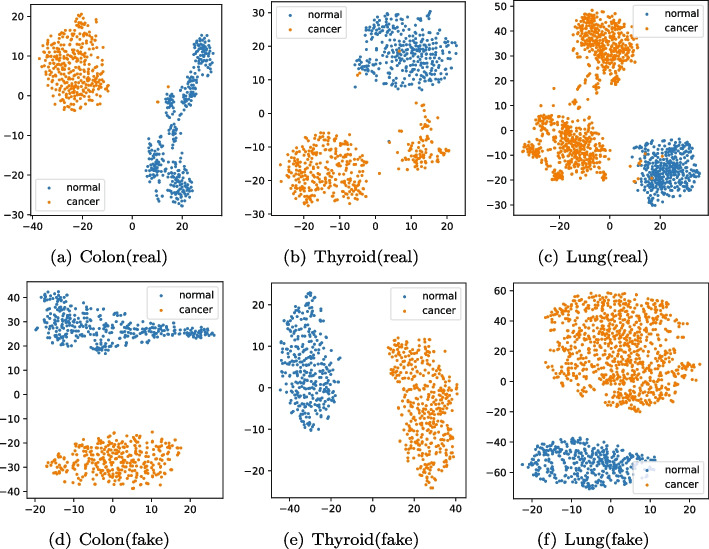
Comparisons of clustering results on the real and the generated samples

Furthermore, in order to intuitively reflect the clustering ability of the samples generated by model MDWGAN-GP, we compare the cluster results on the real samples with those on the generated ones. As shown in Figs. 6 and 7, there datasets such as Colon, Thyroid and Lung were adopted. The dimensionality of each sample was reduced to two with t-SNE [46]. From Fig. 6 we can discover that the generated samples almost overlap with the real ones. Moreover, the clustering results in Fig. 7 demonstrate that the generated samples present better linear separability than the real ones, indicating that it might be better to perform differential analysis between normal and cancer tissues using the generated datasets.

### Ablation experiments

**Fig. 8:**
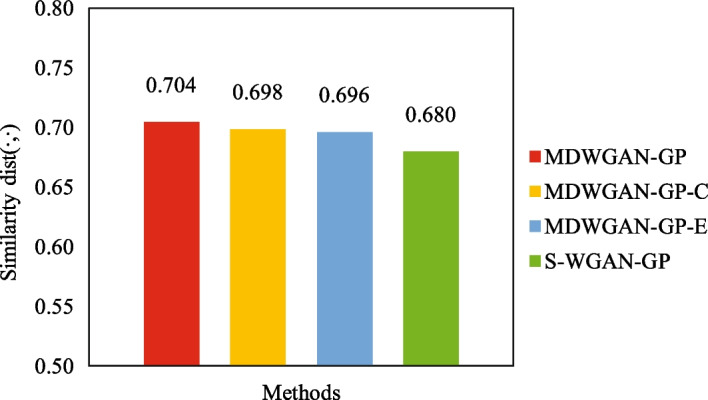
Comparisons of similarity *dist*($\cdot$, $\cdot$) between the real and generated samples

As mentioned before, the training samples were enriched with linear graph convolution in method MDWGAN-GP. Here a series of ablation experiments were conducted on the GT dataset. The training set was constructed by randomly selecting 10% of the samples from each tissue, and the remaining 90% of the samples were chosen as the test set. Figure 8 compares the similarity between the real data and the generated one in terms of *dist*($\cdot$, $\cdot$). In this figure, MDWGAN-GP-C (resp. MDWGAN-GP-E) represents the model adopting only Cosine distance (resp. Euclidean distance). As can be seen from the figure, the MDWGAN-GP model has the highest *dist*($\cdot$, $\cdot$) among the four models. In the subsequent two subsections, experiments were conducted to further test the usability of samples generated with method MDWGAN-GP.

### Comparison of the correlations among key genes

**Fig. 9:**
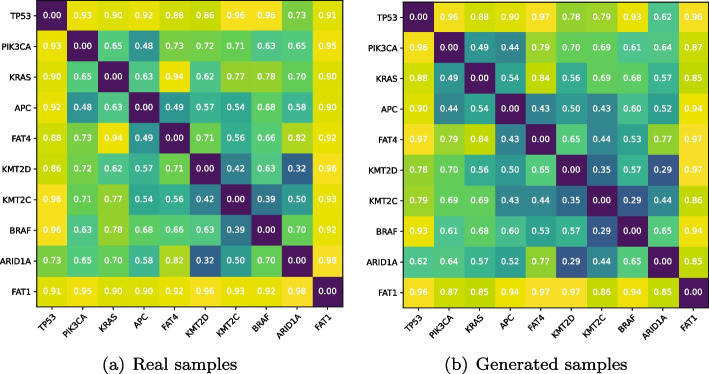
Comparisons of the correlations among 10 key genes

Ten most frequently mutated genes in human cancers [47] were chosen as key genes. The correlations among them are calculated and presented based on the generated and the real expression data, respectively. As can be seen in Fig. 9, a pair of $10 \times 10$ symmetric matrices record the distance $d_{jk}$ (*j*,*k*=1,2,...,10) among the ten key genes. Figure 9a represents the correlations based on the real samples, while Fig. 9b represents those based on the generated samples of model MDWGAN-GP. It can be seen that the distances among genes calculated basing the two different kinds of samples are close, indicating the correlations among genes in the generated data well approximate to those in the real data.

### Comparison of differentially expressed genes (DEGs)

As analyzed above, compared with using the real datasets, it might be better to conduct differential analysis between normal and cancer tissues using the generated ones. In this section, comparisons were further performed between the differentially expressed genes identified based on the generated datasets and those identified based on the real ones. Eighty percent of all pan-cancer samples were randomly selected as the training set, and the same number of samples were generated with model MDWGAN-GP. DESeq2 package of R was called to calculate the difference fold and *p*-value for each gene by using the denormalized generated expression data, and the genes with $|log2(fold \ change)|$ greater than 3 and *p*-values less than 0.05 were selected as differentially expressed genes. For the convenience of description, we use “real-DEGs” and “fake-DEGs” to denote the DEGs ascertained based on the real and the generated datasets, respectively.

As shown in Table 6, for most cancer types, the number of fake-DEGs approximates to that of real-DEGs. Additionally, breast cancer was taken as an example to analyze the association between DEGs and cancers. Firstly, among the top 286 real-DEGs (resp. fake-DEGs), 165 (resp. 177) breast cancer related genes were ascertained basing on the DisGeNET database (v7.0) [48]. It is obvious that the number of breast cancer related DEGs obtained from the generated data are greater than that obtained from the real one.

**Table 6:** Comparisons of the number of differentially expressed genes

Tissue	Real-DEGs	Fake-DEGs	Intersection
Salivary	294	256	176
Uterus	528	438	310
Colon	321	341	239
Prostate	43	12	9
Liver	226	248	158
Bladder	393	451	201
Breast	286	300	203
Stomach	114	138	59
Kidney	270	244	172
Thyroid	134	131	102
Lung	388	375	283
Esophagus_mus	927	798	695

**Fig. 10:**
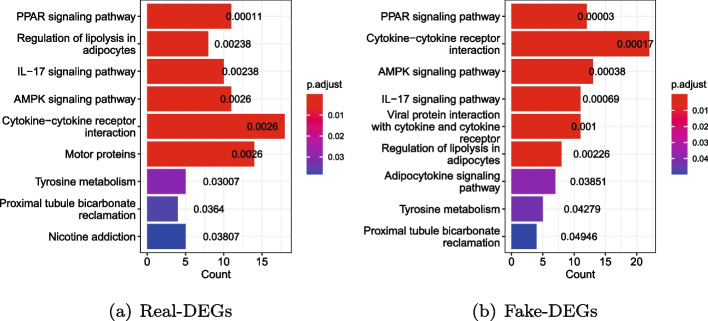
Comparisons of pathways enriched by real-DEGs and fake-DEGs

**Fig. 11:**
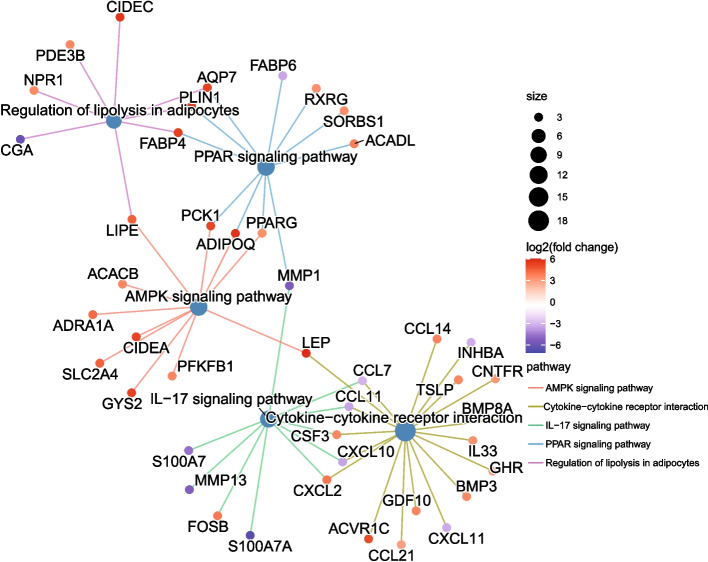
The five top pathways enriched by real-DEGs

**Fig. 12:**
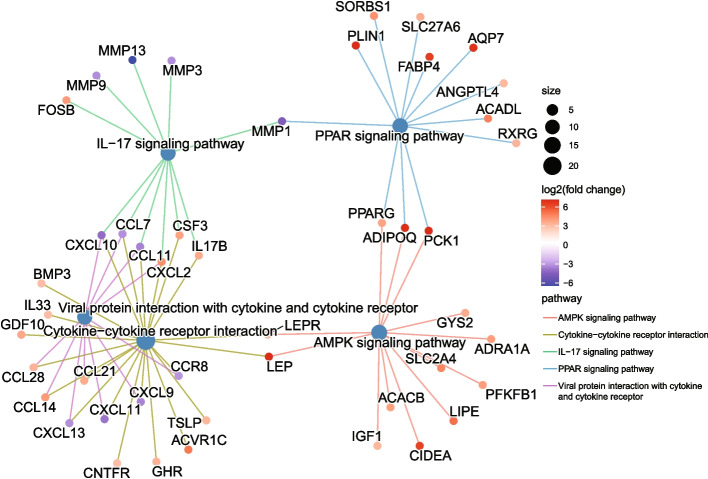
The five top pathways enriched by fake-DEGs

Secondly, package clusterProfiler of R [49] was called to conduct enrichment analysis for the DEGs based on the KEGG database [50]. As displayed in Fig. 10, both real-DEGs and fake-DEGs are enriched in nine biological pathways. The color of bars indicates the degree of significance, and the length of them counts the number of DEGs enriched. Among the two groups of enriched biological pathways, seven breast cancer related pathways are enriched by both real-DEGs and fake-DEGs. The PPAR signaling pathway has been reported as a potential biomarker for the diagnosis of breast cancer [51–53]. Cytokine-cytokine receptor interaction plays an important role in the metastasis of breast cancer and its development [54]. Aberrant AMPK signaling pathways may play a role in the regulation of growth, survival and the development of drug resistance in triple-negative breast cancer [55]. IL-17 signaling pathway has been demonstrated to promote the proliferation, invasion and metastasis of breast cells, and is significantly associated with the poor prognosis of breast patients [56]. Regulation of lipolysis in adipocytes pathway promotes the proliferation and migration of breast cancer cell [57]. Tyrosine metabolism pathway regulates the development of breast cancer [58]. Proximal tubule bicarbonate reclamation pathway indirectly regulates the proliferation of breast cancer cell through TASK-2 [59]. In addition, a pair of breast cancer related biological pathways, i.e., Viral protein interaction with cytokine and cytokine receptor and Adipocytokine signaling pathway, are also enriched by the fake-DEGs. Viral protein interaction with cytokine and cytokine receptor has been reported to be significant for breast cancer [60]. Adipocytokine signaling pathway can mediate the survival, growth, invasion, and metastasis of breast cancer cells through different cellular and molecular mechanisms, thus reducing survival time and contributing to malignancy [61]. Figure 11 (resp. Figure 12) further illustrates the five top pathways enriched by real-DEGs (resp. fake-DEGs) in term of adjusted *p*-values. The steelblue nodes represent the pathways, and the size of which indicates the number of DEGs enriched. Other colored small nodes represent the DEGs, and the color of which indicates its value of $log2(fold \ change)$.

## Conclusions and future directions

Since it is both difficult and expensive for gathering gene expression data with biological experiments, generating them through computational approaches has aroused great attentions. In this study, a generative adversarial network model MDWGAN-GP, having multiple discriminators, is put forward. A novel method is designed for enriching training samples based on linear graph convolutional network. Compared with other state-of-the-art methods, the MDWGAN-GP method can produce higher quality generated gene expression data in most cases. In addition, some critical biomarkers, enriching in some significant biological pathways, are identified based on the generated data. All of these have been verified through extensive experiments performed on real biological data.

However, during the process of experiments, we found that GAN and its improved versions have the inherent defect of being difficult to train. It has been reported that the diffusion model can ensure sample diversity by means of adding and removing noise step by step [62]. It is anticipated to do well in generating high quality and diverse gene expression data, which will be studied in the future.

## Data Availability

The datasets used in this paper and the source code of MDWGAN-GP are available at https://github.com/lryup/MDWGAN-GP.
